# Vitamin D Analogs Can Retard the Onset or Progression of Diabetic Kidney Disease: A Systematic Review

**DOI:** 10.3389/fcdhc.2021.763844

**Published:** 2021-10-15

**Authors:** Samuel N. Uwaezuoke

**Affiliations:** Department of Pediatrics, University of Nigeria Teaching Hospital, Enugu, Nigeria

**Keywords:** Vitamin D, ergocalciferol, therapeutics, diabetic nephropathy, analogs, diabetic kidney disease

## Abstract

**Introduction:**

Previous studies have shown that vitamin D analogs (such as paricalcitol) can reduce albuminuria in patients with diabetes mellitus and retard the progression of diabetic kidney disease (DKD). A recent systematic review reported significant improvement of renal function in patients with DKD who received vitamin D or its analogs. Study-driven data about their use in improving DKD outcomes have continued to accumulate over the years.

**Aim:**

This paper aims to systematically review the contemporary evidence about the effectiveness of vitamin D analogs in retarding the onset or progression of DKD.

**Methods:**

With appropriate descriptors, two electronic databases (PubMed and Google Scholar) were searched for articles published between 2015 and 2021 in the English language. Primary studies that fulfilled the inclusion criteria were selected; their titles and abstracts were screened, and duplicates were removed. Relevant data were retrieved from the final selected studies using a preconceived data-extraction form.

**Results:**

A total of eight studies (three randomized-controlled trials, one prospective study, and four cross-sectional studies) were reviewed. A total of 6,243 participants were investigated in the eight studies and comprised young adults, middle-aged adults, and the elderly with a male-gender predominance. One randomized controlled trial reported that paricalcitol significantly improved renal function in type 1 diabetes patients with renal impairment when combined with renin-angiotensin-aldosterone system (RAAS) blockers. A strong correlation between vitamin D deficiency and DKD risk was noted in the majority of the cross-sectional studies. High doses of cholecalciferol (4,000 or 10,000 IU/day), given early in DKD, significantly reduced disease prevalence.

**Conclusion:**

Paricalcitol may retard the onset or progression of DKD, especially if administered in combination with RAAS blockers. The association of vitamin D deficiency with DKD risk also supports this therapeutic effect. Future systematic reviews are still needed to strengthen the current evidence on therapeutic benefit of vitamin D or its analogs in DKD.

## Introduction

Vitamin D is involved in bone-mineral homeostasis. However, several non-calcemic actions of its bioactive form (calcitriol) are well documented (1–3). These actions are possible because of the wide distribution of vitamin D receptors in the body. The significant effects of calcitriol include modulation of insulin and parathyroid hormone (PTH) secretion, control of cellular proliferation and differentiation, and modulation of immune function (3). Specifically, calcitriol inhibits PTH secretion but facilitates insulin secretion; it prevents cellular proliferation but enhances cellular differentiation. It also inhibits adaptive immunity but stimulates innate immunity (3).

Regarding the effects of calcitriol on the immune system, B-cell proliferation and immunoglobulin production are suppressed, and cell proliferation (especially T-helper-1 cells) is delayed following its inhibitory action on adaptive immunity (4–6). On the other hand, its stimulatory action on innate immunity involves its upregulation of cathelicidin in epithelial and myeloid cells (7), and its inhibition of the synthesis of inflammatory cytokines like interleukin (IL) 1, IL6, IL8, IL12, and tumor necrosis factor-alpha (TNFα) in human monocytes (8). Thus, calcitriol provides a negative- feedback mechanism to mitigate excessive inflammation by interfering with the expression of pro-inflammatory cytokines. These non-calcemic actions have implications for the use of calcitriol or its analogs in attenuating the progression of inflammation-associated diseases such as diabetic kidney disease (DKD).

Different hypotheses on the pathophysiologic mechanisms of DKD have been suggested (9, 10). However, the currently accepted hypothesis assumes that renal fibrosis is the endpoint of a cascade of events arising from hyperglycemia-induced inflammation, oxidative stress, hypoxia, renal hemodynamic changes, and overactive renin-angiotensin-aldosterone system (RAAS) (11). In other words, DKD is a complex disease that arises from the interaction between hemodynamic/metabolic pathways and oxidative stress/inflammatory pathways. In chronic kidney disease (CKD), activation of RAAS and renal inflammation stimulate renal fibrosis and the progression to end-stage kidney disease (ESKD).

DKD shows structural changes in the nephron (mesangial expansion, thickening of the tubules and glomerular basement membrane, and glomerulosclerosis) and functional changes that affect urine albumin excretion, estimated glomerular filtration rate (eGFR), and blood pressure (12). Thus, the clinical staging of DKD (stages 1 to 5) incorporates eGFR, albuminuria, and hypertension; each parameter worsens as the staging-numerical increases (13).

Conventional renoprotective interventions against DKD progression target specific modifiable risk factors, whereas the novel strategies act at specific stages of disease pathogenesis (14). Both conventional interventions (strict glycemic control, control of hypertension, treatment of dyslipidemia, and lifestyle modification) and novel drugs (uric acid antagonists, vitamin D analogs, endothelin receptor antagonists, and glucose-lowering agents) are effective in slowing down the onset of DKD or its progression to ESKD. Animal-experimental data, human observational studies, and short-term clinical trials suggest that vitamin D and omega-3 fatty acid supplements may be safe and cheap pharmacologic interventions to reduce the incidence and progression of DKD (15). For instance, a few previous clinical trials have shown that vitamin D analogs (such as paricalcitol) reduced albuminuria in patients with diabetes mellitus and the progression of its renal complications. They possibly act by inhibiting RAAS and preventing glomerulosclerosis (16, 17). Also, paricalcitol is known to reduce the renal interstitial fibrosis induced by CKD through a synergy of its inhibitory actions on RAAS, inflammation, and epithelial/mesenchymal transition (18). A recent systematic review that evaluated the therapeutic role of cholecalciferol, calcitriol, and paricalcitol in patients with DKD reported significant improvement of their renal functions (19). Thus, the use of vitamin D analogs constitutes a possible new treatment strategy for the prevention of DKD progression.

Following the review published six years ago (19), study-driven data about the use of vitamin D or its analogs in ameliorating DKD outcomes have continued to accumulate to date. Therefore, this paper aims to systematically review the contemporary evidence about the effectiveness of vitamin D analogs in retarding the onset or progression of DKD. The findings will hopefully add to the evidence-based medicine regarding the use of these pharmacologic agents in DKD. The review was conducted and written in conformity with the Preferred Reporting Items for Systematic Reviews and Meta-Analyses (PRISMA) guidelines (20).

## Methods

### Search Strategy

The PubMed and Google Scholar databases were searched for articles published in the last six years, i.e., between 2015 and 2021. (Date of the last search: 9^th^ August 2021). For the Google Scholar database, these were the descriptors in different combinations: ‘type 2 diabetes mellitus’, ‘renal complication’, ‘diabetic kidney disease’, ‘diabetic nephropathy’, ‘vitamin D receptors’, ‘calcitriol’, vitamin D analogs’ and ‘end-stage kidney disease’. For PubMed database, the following descriptors were the search details: ‘vitamin D’, ‘ergocalciferol’, ‘therapeutics’, ‘diabetic nephropathy’ (MeSH) and ‘vitamin D’, ‘ergocalciferol’, ‘analogs’, ‘therapy’ and ‘diabetic kidney disease’ (All search fields).

### Inclusion and Exclusion Criteria

Primary studies that met the following inclusion criteria were selected: (i). randomized control trials or prospective longitudinal studies or cross-sectional studies on human subjects (ii) Full-text studies published in or translated into the English language (iii) Studies whose population or patients were diagnosed with type 1 or type 2 diabetes or DKD; who received vitamin D or its analogs as interventional measures; who were compared with similar subjects given placebo or alternative drugs; and whose outcomes included renal function parameters or other biochemical parameters (i.e., studies that fulfilled the ‘PICO’ acronym – **P**opulation or **P**atients, **I**ntervention, **C**omparators, and **O**utcomes), and (iv) studies that evaluated the association between vitamin D status and DKD, suggesting the therapeutic use of vitamin D or its analogs in the latter. Excluded articles were abstracts, letters to the Editor, reviews, editorials, commentaries, and studies without either primary data or described study methods.

### Study Selection

After screening the titles and abstracts of retrieved published articles from the two electronic databases, potentially eligible full-text articles were evaluated for final inclusion to the list of articles for the present systematic review. Duplicates and articles (whose aims were unrelated to the aim of the present systematic review) were removed during the selection process.

### Quality Assessment

The methodological quality of the selected studies was evaluated using the Newcastle-Ottawa Scale, which assesses non-randomized studies (21). The scale comprises the following criteria for assessing case-control or cross-sectional studies: ‘selection’ (maximum of 5 stars), ‘comparability’ (maximum of 2 stars), and ‘exposure/outcome’ (maximum of 3 stars). The rating of studies was categorized as high if assigned ≥ 7 stars or low if assigned < 7 stars.

### Data Extraction and Data Items

With a preconceived data-extraction form, relevant data were retrieved from the selected studies. The form was designed to gather the first author’s name, year of publication, study setting, country, study design, study population, sample size, and demographic characteristics of patients, such as age and gender distribution. Other extracted data were the diagnosed disease, the pharmacologic interventions (vitamin D or its analogs and placebo or alternative drugs), and the renal function parameters or other biochemical parameters evaluated as outcomes or as parameters of vitamin D status.

### Data Synthesis

Data about the study outcomes were assessed to establish the effectiveness of vitamin D or its analogs in retarding the onset and progression of DKD. Also, qualitative data synthesis was conducted to compare outcomes or endpoints in the interventional group (patients that received vitamin D or its analogs) with the comparative group (patients that received placebo or alternative drugs). The data synthesis was also applied to assessing parameters of vitamin D status in patients with diabetes or DKD.

## Results

### Study Selection

**Figure 1:**
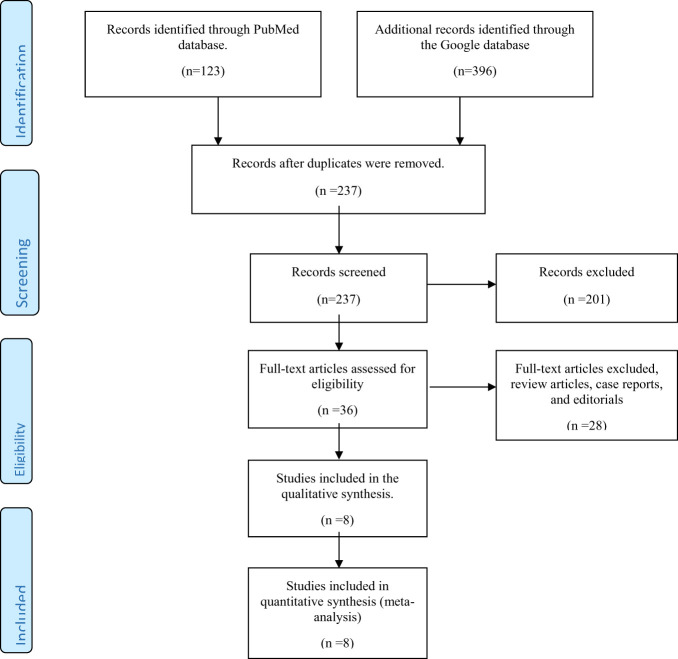
Preferred Reporting Items for Systematic Reviews and Meta-Analyses (PRISMA) flow diagram for inclusion of studies reporting the use of vitamin D or its analogs in diabetic kidney disease.

As shown in **Figure 1**, 123 and 396 relevant papers were identified in the PubMed and Google Scholar databases, respectively, giving 519 papers. Removal of duplicates from both databases scaled down the number of papers to 237. These papers were then screened for their relevance to the present systematic review. Thirty-six papers were left after this initial screening. Further exclusion of 28 papers (including reviews, case reports, and editorials) yielded eight full-text original articles assessed for eligibility according to the inclusion criteria. These eight articles were selected for qualitative analysis in the present systematic review.

### Study Characteristics

The eight selected full-text original articles comprise three randomized controlled trials (22–24), a short-duration prospective study (25), and four cross-sectional studies (26–29). The countries where these studies were conducted are located in North America (23, 24), South America (25, 26),Asia (27–29), and Europe (22). All the studies were hospital-based. A total of 6,243 subjects participated in the eight studies. The participants’ age distribution varied in six studies with mean ages of 28.6± 8.1 years (26), 29.0 ± 8.1 years (25), 50.8 ± 7.6 years (24), 55.2± 10.2 years (27), 57 ± 9 years (22), and 61.6± 10.9 years (29). One study reported a median age of 64 years (age range: 53-71years) in the intervention group and 61 years (age range: 51-71 years) in the placebo group (23). Additionally, their gender distribution shows that both sexes were equally distributed in one study (25), with female predominance in one study (26), and male predominance in the majority of the studies (22–24, 27–29). Using the Newcastle-Ottawa Scale, the methodological quality of four cross-sectional studies reveals a star-rating of < 7 (low quality) for one study (29), and ≥ 7 (high quality) for the rest of the studies (26–28). Also, a rating of < 7 applies to the prospective study (25), and ≥ 7 to the three randomized controlled trials (22–24) (**Table 1**).

**Table 1:** Characteristics of studies that reported an association between vitamin D or its analogs and diabetes mellitus or diabetic kidney disease. .

Study (first author’s name and year of publication)	Country of study	Study setting	Study population (sample size and age/gender distribution)	Study design
**Joergensen et al., 2015 (** 22 **)**	Denmark	Steno Diabetes Centre, Gentofte	48 participants Mean age: 57 ± 9 years M/F:32/16	A double-blind, randomized, placebo-controlled crossover trial
**Thethi et al., 2015 (** 23 **)**	United States of America	Tulane University Health Sciences Centre, New Orleans, LA	27 participants (intervention group) & 28 participants (placebo group) Median age: 64, 53-71 years (intervention group) & 61, 51-71 years (placebo group) M/F: 19/8 (intervention group) & 18/10 (placebo group)	Double-blind, randomized placebo-controlled trial
**Felício et al., 2016 (** 26 **)**	Brazil	Federal University of Pará	73 participants (37 patients & 36 controls) Mean age of patients: 28.6 ± 8.1 years (M/F:13/24) Mean age of controls: 25.2 ± 3.0 years (M/F:19/17)	Cross-sectional, comparative study
**Felício et al., 2017 (** 25 **)**	Brazil	Federal University of Pará	22 participants Mean age: 29.0 ± 8.1 years M/F:11/11	3-month prospective study
**Zaheer et al., 2018 (** 24 **)**	United States of America	Brigham and Women’s Hospital, Harvard Medical School	18 participants Mean age: 50.8 ± 7.6 years M/F:11/7	Randomized, double-blinded, and placebo-controlled study
**Xie et al., 2019 (** 27 **)**	China	Jiangsu Province Hospital on Integration of Chinese and Western Medicine	351 participants Age range: 25-75 years (mean age: 55.2 ± 10.3 years) M/F:242/109	Cross-sectional study
**Xiao et al., 2020 (** 28 **)**	China	Second Xiangya Hospital of Central South University	4284 participants Age: ≥ 18 years (young adults- 12.5%, middle age- 57.0%, elderly- 30.5%)* M/F:52.6%/47.4%	Cross-sectional study
**Hong et al, 2021 (** 29 **)**	South Korea	Eulji Medical Centre, Eulji University School of Medicine, Seoul & Ewha Womans University School of Medicine, Seoul	1392 participants Mean age: 61.6 ± 10.9 years M/F: 57.9%/42.1%	Cross-sectional, retrospective study

M, male; F, female; *young adults (18-44 years), middle-aged adults (45-64 years), elderly (≥65 years).

### Study Findings

The following significant findings were reported in each of the eight studies (**Table 2**). Among the cross-sectional studies (26–29), Felício et al. evaluated the association between low levels of vitamin D and the presence and degree of DKD in T1DM patients (26). They estimated the levels of 25-hydroxyvitamin D and albuminuria in 37 T1DM patients with normal glomerular filtration rate (GFR), i.e.,> 90 ml/min/1.73m^2^ and 36 controls with normal serum creatinine. Patients with T1DM and hypovitaminosis D had significantly higher levels of albuminuria compared to their counterparts with normal vitamin D levels [albuminuria (log_10_) = 1.92 *versus* 1.44; p<0.05] (26). Also, based on the DKD stage, there were lower levels of 25-hydroxyvitamin D in the macroalbuminuric group compared to the normoalbuminuric and microalbuminuric groups. In the study by Xie et al. (27), the association between 25-hydroxyvitamin D level and DKD in 315 patients with T2DM was assessed. The investigators estimated these patients’ serum 25-hydroxyvitamin D and urine albumin-to-creatinine ratio (UACR) levels. They reported a significantly higher prevalence of 25-hydroxyvitamin D deficiency in patients with microalbuminuria than the prevalence in those with normoalbuminuria (25.1% *versus* 19.6%; p<0.05), and the highest prevalence of the vitamin deficiency in the macroalbuminuria group (37.8%; p<0.01) (27). Furthermore, after logistic regression analyses, low 25-hydroxyvitamin D levels were shown to be associated with DKD [odds ratio (OR) *=* 1.51, 95% confidence interval (CI) 1.16–1.97]. The association even appeared more robust after the authors adjusted for gender, hypertension, elevated systolic blood pressure, glycemic status, and hyperuricemia (OR = 1.62, 95% CI 1.19–2.20) (27). Xiao et al. investigated the association between vitamin D deficiency and diabetic vascular complications, including diabetic retinopathy, DKD, and diabetic foot ulcers (28). They studied a large sample size of 4,284 Chinese patients with T2DM. After estimating serum 25-hydroxyvitamin D levels for the participants’ vitamin D status, they found a significant association of vitamin D deficiency with diabetic foot ulcers but not with DKD or diabetic retinopathy. Specifically, the prevalence ratios (95% CI) of vitamin D deficiency in patients with diabetic retinopathy, DKD, and diabetic foot ulcers were 1.093 (0.983-1.215), 1.041 (0.937-1.156), and 1.656 (1.159-2.367) respectively (after adjusting for demographic and clinical variables) (28). Their findings suggest a significant association of vitamin D deficiency with diabetic foot ulcer but not with DKD or diabetic retinopathy. Finally, the cross-sectional retrospective study by Hong et al. evaluated the relationship between vitamin D status and metabolic parameters and T2DM complications in a reasonably large sample size of 1,392 patients with T2DM (29). The authors conducted a retrospective evaluation of glycated hemoglobin (HbA1c), lipid profile, liver and kidney function, and UACR, and measurement of serum 25-hydroxyvitamin D level in these patients. They reported these significant findings: independent association of vitamin D deficiency with nephropathy after adjusting for confounders, a positive correlation of 25-hydroxyvitamin D level with high-density lipoprotein-cholesterol (HDL-C) level, and its negative correlation with HbA1c value, triglyceride (TG) level, and UACR, as well as a significant association of HDL-C and UACR with 25-hydroxyvitamin D deficiency (29).

**Table 2:** Significant findings of the studies on the association between vitamin D or its analogs and diabetes mellitus or diabetic kidney disease.

Study (first author’s name and year of publication)	Study aims	Study interventions	Study outcomes or endpoints	Major findings
**Joergensen et al., 2015 (** 22 **)**	To evaluate the effects of therapy with paricalcitol on markers of cardiovascular risk and kidney function in patients with T1DM and DN	Twelve weeks of paricalcitol (1-2μg daily) and 12 weeks of placebo therapy on 48 participants with T1DM and DN receiving RAAS blockers and diuretics	-Plasma N-terminal proBNP -Urinary albumin excretion rate	-No significant effect on plasma N-terminal proBNP concentration -Significant reduction of urinary albumin excretion rate by 18% and eGFR by 5 ml/min/1.73m (2)
**Thethi et al., 2015 (** 23 **)**	To compare the effects of paricalcitol and placebo on endothelial function and inflammation and oxidative stress markers in patients with T2DM and CKD (stage 3 or 4).	Once-daily oral administration of paricalcitol (1μg) or placebo for three months	-Brachial artery FMD & NMD -Biomarkers of inflammation & oxidative stress	- No significant difference in FMD, NMD, or biomarkers after paricalcitol or placebo treatment.
**Felício et al., 2016 (** 26 **)**	To evaluate the association between low levels of vitamin D and the presence and degree of DKD in T1DM	Estimation of levels of 25-hydroxyvitamin D and albuminuria in patients and controls	-25-hydroxyvitamin D level -Albuminuria level	-Significantly higher levels of albuminuria in patients with T1DM & hypovitaminosis D -Negative correlations between vitamin D levels and both albuminuria & DKD stages**^†^**
**Felício et al., 2017 (** 25 **)**	To evaluate reduction of albuminuria in patients with T1DM supplemented with a high dose of vitamin D	Administration of 4,000 and 10,000 IU/day of cholecalciferol for 12 weeks	-UACR before and after vitamin D supplementation.	-Significant reduction of DKD prevalence at the end of the study -Improvement in DKD stage among T1DM patients with DKD at the commencement of the study
**Zaheer et al., 2018 (** 24 **)**	To investigate the effect of direct VDR activation with calcitriol on circulating RAAS activity and vascular hemodynamics in T2DM.	Administration of calcitriol or placebo on patients with well-controlled T2DM without CKD	-PRA -Serum and urine aldosterone -RPF -MAP	-Significant elevation of 1,25 dihydroxy vitamin D with calcitriol but no change with placebo -No significant differences in PRA, serum or urinary aldosterone, MAP & RPF between both interventions
**Xie et al., 2019 (** 27 **)**	To assess the association between 25-hydroxyvitamin D level and DKD in patients with T2DM	Estimation of serum 25 hydroxyvitamin D levels & UACR levels^§^	-Serum 25 hydroxyvitamin D -UACR	-Highest prevalence of 25 hydroxyvitamin D insufficiency in patients with macroalbuminuria -25 hydroxyvitamin D insufficiency found to be associated with DKD after logistic regression analyses
**Xiao et al., 2020 (** 28 **)**	To investigate the association between vitamin D deficiency and diabetic vascular complications, including diabetic retinopathy, DKD, and diabetic foot ulcers	Estimation of serum 25 hydroxyvitamin D levels to assess vitamin D deficiency	-Serum 25 hydroxyvitamin D	-Significant association of vitamin D deficiency with diabetic foot ulcer but not with DKD or diabetic retinopathy
**Hong et al., 2021 (** 29 **)**	To evaluate the relationship between vitamin D status and metabolic parameters and T2DM complications	Retrospective evaluation of HbA1c, lipid profile, liver & kidney function, and UACR Measurement of serum 25 hydroxyvitamin D level	-Serum 25 hydroxyvitamin D -UACR -Lipid profile -HbA1c	-Vitamin D deficiency independently related to nephropathy after adjusting to confounders -25 hydroxyvitamin D level positively correlated with HDL-C level and negatively correlated with HbA1c, TG level & UACR -HDL-C & UACR significantly associated with 25 hydroxyvitamin D deficiency

T1DM, type 1 diabetes mellitus; T2DM, type 2 diabetes mellitus; CKD, chronic kidney disease; DKD, diabetic kidney disease; DN, diabetic nephropathy; RAAS, renin-angiotensin-aldosterone system; proBNP, probrain natriuretic peptide; eGFR, estimated glomerular filtration rate; FMD, flow-mediated dilatation; NMD, nitroglycerine-mediated dilatation; VDR, vitamin D receptor; PRA, plasma renin activity; RPF, renal plasma flow; MAP, mean arterial pressure; UACR, urine albumin-to-creatinine ratio; HbA1c, glycated hemoglobin; HDL-C, high density lipoprotein-cholesterol; TG, triglyceride.

**^†^** normo, micro, and macroalbuminuria ^§^normoalbuminuria (UACR: < 30 mg/g), microalbuminuria (UACR: 30-300 mg/g), macroalbuminuria (UACR:≥ 300 mg/g).

In the 3-month prospective study (pilot study) by Felício et al., 4,000 and 10,000 IU/day of cholecalciferol were administered for 12 weeks on 22 patients with T1DM (25). The investigators evaluated reduction of albuminuria in these patients after supplementation with the high doses of vitamin D. After estimating the UACR before and after vitamin D supplementation, they reported a significant reduction of DKD prevalence at the end of the study and improvement in DKD stage among the patients with DKD at the commencement of the study. However, being a pilot study, the small sample precluded the generalizability of the study findings.

The three randomized controlled trials show variable findings regarding the effects of vitamin D or its analog on patients with T1DM or T2DM and DKD. For instance, Joergensen et al. evaluated the effects of therapy with paricalcitol on cardiovascular risk and kidney function markers in patients with T1DM and diabetic nephropathy (DN) (22). They administered paricalcitol (1-2μg daily) and placebo therapy for twelve weeks on 48 patients with T1DM and DN receiving RAAS blockers and diuretics. The primary and secondary endpoints were changes in plasma N-terminal pro-brain natriuretic peptide (proBNP) and urinary albumin excretion rate obtained before and after each intervention. Paricalcitol therapy had no significant effect on plasma N-terminal proBNP level (p = 0.6) compared with placebo therapy. However, urinary albumin excretion rate was decreased by 18% (p = 0.03 for comparison), estimated glomerular filtration rate (eGFR) was reduced by 5 ml/min/1.73 m^2^ (p < 0.001) and measured glomerular filtration rate (GFR) was reduced by 1.5 ml/min/1.73 m^2^ (p= 0.2) (22). These findings indicate that paricalcitol therapy did not affect plasma N-terminal proBNP level in patients with T1DM and DN. However, the urinary albumin excretion rate was significantly reduced. On the other hand, Thethi et al. compared the effects of paricalcitol and placebo on endothelial function and inflammation and oxidative stress markers in patients with T2DM and CKD (stage 3 or 4) (23). They administered once-daily oral paricalcitol (1μg) on 27 patients and placebo on 28 patients for three months. Using brachial artery flow-mediated dilatation (FMD) and nitroglycerine-mediated dilatation (NMD) as well as inflammation and oxidative stress biomarkers as baseline and endpoint parameters, the authors demonstrated no significant difference in alteration in FMD, NMD, or these biomarkers after paricalcitol or placebo therapy. The findings were partly attributed to the advanced CKD in the study patients. Finally, Zaheer et al. investigated the effect of direct vitamin D-receptor activation with calcitriol on circulating RAAS activity and vascular hemodynamics in 18 patients with T2DM (24). They administered calcitriol or placebo as interventions on these patients who had well-controlled T2DM without CKD. Plasma renin activity (PRA), serum and urinary aldosterone, renal plasma flow (RPF), and mean arterial pressure (MAP) were used as study endpoints. They found significant elevation of 1, 25-dihydroxy vitamin D with calcitriol but no change with placebo; there were no significant differences in PRA, serum or urinary aldosterone, MAP, and RPF between both interventions (24).

## Discussion

Medical literature is now replete with evidence-based data supporting the relationship between vitamin D status and DKD. By inference, the data underscore the renoprotective property of vitamin D or its analogs in DKD (15–17, 30–35). Although some studies had earlier reported inconclusive findings (30, 36, 37), a systematic review published six years ago (noted to be the first of such a review) indicate the therapeutic role of vitamin D and its analogs in DKD (19). The apparent non-unanimity of research findings on the subject buttresses the need for repeat reviews of current and emerging published data until a strong consensus is reached.

Nevertheless, the potential mechanisms underpinning the renoprotective effects of vitamin D are well described. Vitamin D receptors are highly expressed in the kidney, making the organ a major target site of vitamin D actions. Vitamin D primarily acts as an inhibitor of intrarenal RAAS activation, which occurs due to the effect of diabetes-induced hyperglycemia on the kidney. The RAAS pathway cascades through a sequence of events whose sequelae include renal fibrosis, glomerulosclerosis, inflammation, podocyte injury, endothelial dysfunction, and disruption of the glomerular filtration barrier. The latter contributes to various degrees of albuminuria seen in DKD. These pathological features constitute the essential components of DKD. In vitamin D deficiency, RAAS activation arising from hyperglycemia is unregulated: thus creating the pathogenic pathway for DKD.

**Figure 2:**
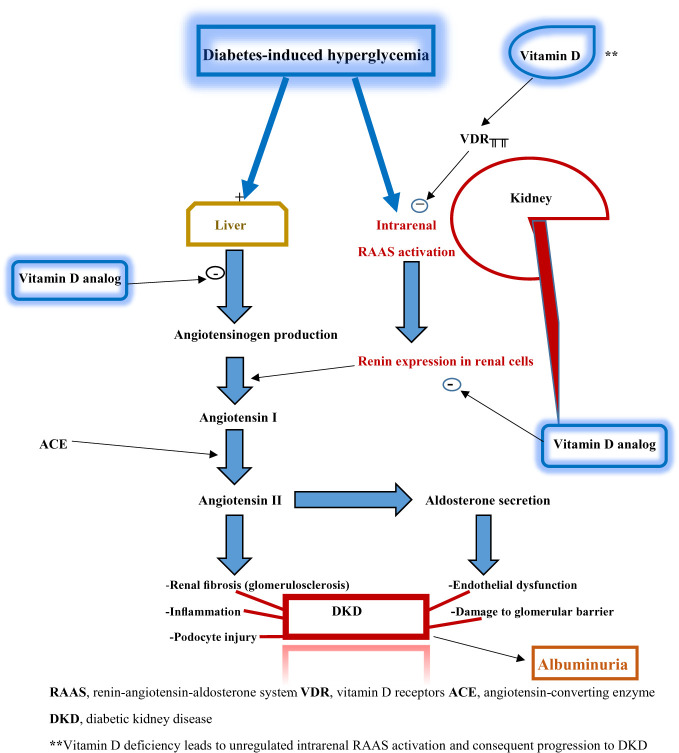
Schematic diagram illustrating the relationship between vitamin D deficiency and diabetic kidney disease.

Additionally, vitamin D analogs (such as paricalcitol) block angiotensinogen production and renin expression in renal cells (**Figure 2**). Thus, vitamin D’s or its analogs’ renoprotective effects are mediated through their actions at different target sites of the hyperglycemia-triggered RAAS pathway. These renoprotective effects can therefore be adjudged as glucose-lowering dependent. Strict glycemic control and glucose-lowering agents (such as sodium-glucose transporter 2 inhibitors, glucagon-like peptide-1 analogs, and dipeptidyl peptidase 4 inhibitors) enhance or synergize with the renoprotective activity of vitamin D (14).

In the present systematic review, data from a total of 121 participants in three randomized- controlled trials suggest that paricalcitol significantly improves renal function in T1DM patients with renal impairment in synergy with RAAS blockade (22), but did not alter endothelial function or biomarkers of inflammation and oxidative stress in T2DM patients with advanced CKD (23). In well-controlled T2DM patients without renal impairment, calcitriol (in comparison with placebo) did not affect markers of RAAS activity and vascular hemodynamics (24). This observation buttresses the fact that vitamin D’s renoprotective effect depends on lowered glucose levels. However, most cross-sectional studies that investigated the association of vitamin D status with diabetes or its complications in a pooled number of 1,816 subjects indicate a strong correlation between vitamin D deficiency and DKD risk (26, 27, 29). A prospective pilot study demonstrated a significant reduction in DKD prevalence with the administration of high doses of cholecalciferol (4,000 or 10,000 IU/day) (25). By extrapolation, these observations underscore the possible therapeutic effects of vitamin D or its analogs in DKD. Most of this review’s findings are in keeping with those of a previous systematic review that analyzed six studies reporting the effect of cholecalciferol, calcitriol, or paricalcitol in patients with DN (19). In this previously published review, three studies (one randomized controlled trial, one non-randomized controlled trial, and one uncontrolled trial) investigated the effect of cholecalciferol (38–40). Additionally, two studies (one randomized-controlled trial and one uncontrolled trial) evaluated the effect of calcitriol (35, 41).

In contrast, one study (a randomized controlled trial) assessed the effect of paricalcitol (17). A summary of the findings from the six studies indicates that vitamin D analog (paricalcitol) and high-dose cholecalciferol showed significant beneficial effects on renal functions (e.g., UACR and eGFR): with high-dose cholecalciferol showing these benefits early in the therapy, which was however not sustained towards the end of the interventions (19). Most findings from the present systematic review are at variance with those of three previous studies (30, 36, 37). The first study showed that impaired vitamin D metabolism is not associated with early GFR reduction or development of hypertension (30). The second study reported that severe vitamin D deficiency independently predicted all-cause mortality rather than renal microvascular complications (36). The third study observed that diabetic patients with persistent microalbuminuria showed no significant difference in surrogate markers of vitamin D metabolism in either the control group or the group of diabetic patients without persistent microalbuminuria (37).

Although differences and flaws in study methods can explain disparities in these study findings, there is difficulty in evaluating the association between low vitamin D levels and DKD. Renal impairment per se can cause vitamin D deficiency: creating the dilemma of ascertaining if the vitamin deficiency is a trigger or a result of DKD progression (26). Nevertheless, hypovitaminosis D is associated with increased urinary albumin excretion and cardiovascular morbidity and mortality in CKD patients in the general population (42, 43). Nuclear receptors in the body, such as vitamin D receptors, negatively regulate inflammation, oxidative stress, and fibrosis, as previously stated: which constitute the hallmark of DKD pathogenesis. Experimental animal studies have demonstrated a higher expression of renin, angiotensin, and angiotensin receptors in murine models with knocked-out vitamin D receptors under diabetic conditions (44). They also show the evolution of severe renal damage leading to early-onset albuminuria, glomerulosclerosis, and interstitial fibrosis due to intra-renal RAAS activation (45). Findings from human observational studies confirm that vitamin D deficiency is associated with both renal impairment and progression of DKD (46, 47). This incontrovertible relationship has led to the initiation of more recent protocols for randomized-control trials, such as evaluating the effect of adding calcitriol to RAAS-blockade therapy on urinary albumin excretion in patients with T2DM (48). The authors’ proposed study is based on the hypothesis that an active vitamin D analog works synergistically with angiotensin-converting enzyme inhibitors (ACEI) or angiotensin-receptor blockers (ARB) to reduce albuminuria in DKD. Previous reports, as stated above, have shown that paricalcitol in combination with RAAS blockade significantly improved renal function in T1DM patients with renal impairment (22). At the same time, calcitriol did not alter the markers of RAAS activity and vascular hemodynamics in T2DM patients who had no renal impairment (24).Given these findings, it appears that bioactive vitamin D or the analog would be more effective in DKD when combined with pharmacologic antagonists of RAAS. The argument that tends to favor this observation is based on the fact that RAAS activation resulting in renal injury occurs more when vitamin D receptors (as negative modulators of inflammation, oxidative stress, and fibrosis) are inhibited in diabetes (44, 45). This inhibition of vitamin D receptors in diabetes is yet another evidence that the renoprotective action of vitamin D may be dependent on normoglycemia.

Again, this systematic review has shown that the dosing and timing of cholecalciferol may predict its effectiveness in ameliorating renal dysfunction in DKD. For instance, a randomized-controlled trial reported that using 2 μg of paricalcitol improved residual albuminuria in patients with T2DM and DKD. However, a reduction of albuminuria was not demonstrated in patients receiving lower doses of paricalcitol at 1 μg daily and those receiving placebo (17). Besides, a meta-analysis of four interventional studies (that used either calcitriol or cholecalciferol in patients with T2DM to reduce proteinuria) reported some interesting findings (49). First, variable renal function outcomes were obtained as only one study documented a reduction of proteinuria (35). In contrast, the remaining three studies showed non-reduction of urinary protein excretion (38, 39, 50). The disparate findings were because of the inclusion of patients with several stages of CKD in these studies. Importantly, the findings of this meta-analysis re-echo those of the previous systematic review (19), because they included three similar studies (35, 38, 39). Nonetheless, two facts stand out: a more favorable outcome from vitamin D supplementation when given in the early stages of CKD/DKD and a greater effectiveness with higher doses of the vitamin or its analogs. Thus, the benefit of this therapeutic strategy needs to be resolved by further research.

The present systematic review has some limitations. The literature search strategy was limited to two electronic databases (PubMed and Google Scholar databases). Although the search-net should have been extended wider, the choice of these two databases was based on the easy accessibility to non-subscription-based full-text original articles. Only a few interventional studies (three randomized-controlled trials) on the topic were published within the past six years. Therefore, additional databases would probably not have made much difference. Secondly, a meta-analysis was not conducted because only three randomized-controlled trials were included. Finally, quality assessment of the included studies did not give details on the various categories of bias that might have arisen during recruitment of the sample population (selection bias), the conduct of the trials (performance bias), and analysis of the results (attrition bias). Despite the randomized-controlled trials, the present review used the Newcastle-Ottawa Scale (for non-randomized studies, case-control, and cross-sectional studies). Apart from the star-rating that encompasses major criteria covering the above details in the likely biases, the criteria were also considered applicable to randomized-controlled trials.

## Conclusions

This systematic review has shown that paricalcitol may retard the onset or progression of DKD, especially when administered in the early stages of the disease and combined with RAAS blockers. The association of vitamin D deficiency with DKD risk reported in the systematically-reviewed cross-sectional studies also lends credence to the role of vitamin D or its analogs in preventing the onset and progression of DKD. Finally, it is essential to resolve if giving higher doses of vitamin D supplementation (4,000 or 10,000 IU/day) is more effective in improving renal outcomes in DKD. The few number and small sample sizes of the reviewed randomized-controlled trials underscore the need to conduct similar studies on more extensive and more representative population samples. Thus, repeat systematic reviews on more randomized-controlled trials are required to strengthen the current evidence on the therapeutic benefit of vitamin D or its analogs in DKD.

## Data Availability Statement

The original contributions presented in the study are included in the article/supplementary material. Further inquiries can be directed to the corresponding author.

## Author Contributions

The author confirms being the sole contributor of this work and has approved it for publication.

## Conflict of Interest

The author declares that the research was conducted in the absence of any commercial or financial relationships that could be construed as a potential conflict of interest.

## Publisher’s Note

All claims expressed in this article are solely those of the authors and do not necessarily represent those of their affiliated organizations, or those of the publisher, the editors and the reviewers. Any product that may be evaluated in this article, or claim that may be made by its manufacturer, is not guaranteed or endorsed by the publisher.

## References

[1] Li YC . Vitamin D Regulation of the Renin-Angiotensin System. J Cell Biochem (2003) 88:327–31. doi: 10.1002/jcb.10343 12520534

[2] Deluca HF . Overview of General Physiologic Features and Functions of Vitamin D. Am. J Clin Nutr (2004) 80:S1689–96. doi: 10.1093/ajcn/80.6.1689S 15585789

[3] Bikle D . Non-Classic Actions of Vitamin D. J Clin Endocrinol Metab (2009) 94:26–34. doi: 10.1093/ajcn/80.6.1689S 18854395 PMC2630868

[4] Rigby WF Stacy T Fanger MW . Inhibition of T Lymphocyte Mitogenesis by 1, 25-Dihydroxy Vitamin D3 (Calcitriol). J Clin Invest (1984) 74:1451–5. doi: 10.1172/JCI111557 PMC425314 6332829

[5] Chen S Sims GP Chen XX Gu YY Chen S Lipsky PE . Modulatory Effects of 1, 25-Dihydroxy Vitamin D3 on Human B-Cell Differentiation. J Immunol (2007) 179:1634–47. doi: 10.4049/jimmunol.179.3.1634 17641030

[6] Lemire JM Archer DC Beck L Spiegelberg HL . Immunosuppressive Actions of 1, 25-Dihydroxy Vitamin D3: Preferential Inhibition of Th 1 Functions. J Nutr (1995) 125:S1704–8. doi: 10.1093/jn/125.suppl_6.1704S 7782931

[7] Gombart AF Borregaard N Koeffler HP . Human Cathelicidin Antimicrobial Peptide (CAMP) Gene is a Direct Target of the Vitamin D Receptor and is Strongly Up-Regulated in Myeloid Cells by 1, 25-Dihydroxy Vitamin D3. FASB J (2005) 19:1067–77. doi: 10.1096/fj.04-3284com 15985530

[8] Almerighi C Sinistro A Cavazza A Ciaprini C Rocchi G Bergamini A . 1α, 25-Dihydroxy Vitamin D3 Inhibits CD4OL-Induced Pro-Inflammatory and Immunomodulatory Activity in Human Monocytes. Cytokine (2009) 45:190–7. doi: 10.1016/j.cyto.2008.12.009 19186073

[9] Dugan LL You Y Ali SS Diamond-Stanic M Miyamoto S DeCleves A . AMPK Dysregulation Promotes the Diabetes-Related Reduction of Superoxide and Mitochondrial Function. J Clin Invest (2013) 123:4888–99. doi: 10.1172/JCI66218 PMC3809777 24135141

[10] Brownlee M . Biochemistry and Molecular Cell Biology of Diabetic Complications. Nature (2001) 414:813–20. doi: 10.1038/414813a 11742414

[11] Lin Y Chang Y Yang S Wu K Chu T . Update of Pathophysiology and Management of Diabetic Kidney Disease. J Formosan Med ASSOC (2018) 117:662–75. doi: 10.1016/j.jfma.2018.02.007 29486908

[12] Satirapoj B Adler SG . Prevalence and Management of Diabetic Nephropathy in Western Countries. Kidney Dis (2015) 1:61–70. doi: 10.1159/000382028 PMC4934803 27536666

[13] Tuttle K . Back to the Future: Glomerular Hyperfiltration and the Diabetic Kidney. Diabetes (2017) 66:14–6. doi: 10.2337/dbi16-0056 PMC5204314 27999101

[14] Uwaezuoke SN Ayuk AC . Diabetic Kidney Disease in Childhood and Adolescence: Conventional and Novel Renoprotective Strategies. EMJ Nephrol (2020) 8:68–77.

[15] de Boer IH Zelnick LR Lin J Schaumberg D Wang L Ruzinski J . Vitamin D and Omega-3 Trial to Prevent and Treat Diabetic Kidney Disease: Rationale, Design, and Baseline Characteristics. Contemp Clin Trials (2018) 74:11–7. doi: 10.1016/j.cct.2018.09.014 PMC6203639 30282055

[16] Agarwal R Acharya M Tian J Hippensteel RL Melnick JZ Qui P . Anti-Proteinuric Effect of Oral Paricalcitol in Chronic Kidney Disease. Kidney Int (2005) 68:2823–8. doi: 10.1111/j.1523-1755.2005.00755.x 16316359

[17] de Zeeuw D Agarwal R Amdahl M Audhya P Coyne D Garimella T . Selective Vitamin D Receptor Activation With Paricalcitol for Reduction of Albuminuria in Patients With Type 2 Diabetes (VITAL Study): A Randomized Controlled Trial. Lancet (2010) 376:1543–51. doi: 10.1016/S0140-6736(10)61032-X 21055801

[18] Martinez-Arias L Panizo S Alonso-Montes C Martin-Virgala J Martin-Carro B Fernandez-Villabrille S . Effects of Calcitriol and Paricalcitol on Renal Fibrosis in CKD. Nephrol Dial Transplant (2021) 36:793–803. doi: 10.1093/ndt/gfaa373 33416889

[19] Chokhandre MK Mahmoud MI Hakami T Jafer M Inamdar AS . Vitamin D & its Analogs in Type 2 Diabetic Nephropathy: A Systematic Review. J Diabet Metab Disord (2015) 14:58. doi: 10.1186/s40200-015-0186-6 PMC4502529 26180775

[20] Moher D Liberati A Tetzlaff J Altman DG the PRISMA Group . Preferred Reporting Items for Systematic Reviews and Meta-Analyses: The PRISMA Statement. PLoS Med (2009) 6:e1000097. doi: 10.1371/journal.pMed1000097 19621072 PMC2707599

[21] Wells G Shea B O’Connell D . The Newcastle-Ottawa Scale (NOS). Disponibile all’indirizzo (2000). Available at: http://www.ohri.ca/programs/clinical_epidemiology/oxford.

[22] Joergensen C Tarnow L Goetze JP Rossing P . Vitamin D Analog Therapy, Cardiovascular Risk and Kidney Function in People With Type 1 Diabetes Mellitus and Diabetic Nephropathy: A Randomized Trial. Diabet Med (2015) 32:374–81. doi: 10.1111/dme.12606 25307511

[23] Thethi TK Bajwa MA Ghanim H . Effect of Paricalcitol on Endothelial Function and Inflammation in Type 2 Diabetes and Chronic Kidney Disease. J Diabetes Complications (2015) 29:433–7. doi: 10.1016/j.jdiacomp.2015.01.004 PMC4392813 25633573

[24] Zaheer S Taquechel K Brown JM Adler GK Williams JS Vaidya A . A Randomized Intervention Study to Evaluate the Effect of Calcitriol Therapy on the Renin-Angiotensin System in Diabetes. J Renin Angiotensin Aldosterone Syst (2018) 19:1–8. doi: 10.1177/1470320317754178 PMC5896865 29562806

[25] Felício JS de Oliveira AF Peixoto AS Souza ACCB Abrahao Meto JF de Melo FTC . Albuminuria Reduction After High Dose of Vitamin D in Patients With Type 1 Diabetes Mellitus: A Pilot Study. Front Endocrinol (2017) 8:199. doi: 10.3389/fendo.2017.00199 PMC5557778 28855892

[26] Felício JS Luz RM de Melo FTC de Souza Resende F de Oliveira AF Peixoto AS . Vitamin D on Early Stages of Diabetic Kidney Disease: A Cross-Sectional Study in Patients With Type 1 Diabetes Mellitus. Front Endocrinol (2016) 7:149. doi: 10.3389/fendo.2016.00149 PMC5151807 28018288

[27] Xie S Huang L Cao W Hu Y Sun H Cao L . Association Between Serum 25-Hydroxyvitamin D and Diabetic Kidney Disease in Chinese Patients With Type 2 Diabetes. PLoS One (2019) 14:e0214728. doi: 10.1371/journal.pone.0214728 31017918 PMC6481913

[28] Xiao Y Wei L Xiong X Yang M Sun L . Association Between Vitamin D Status and Diabetic Complications in Patients With Type 2 Diabetes Mellitus: A Cross-Sectional Study in Hunan China. Front Endocrinol (Lausanne) (2020) 11:564738. doi: 10.3389/fendo.2020.564738 33042022 PMC7525149

[29] Hong S Kim YB Choi HS Jeong T Kim JT Sung YA . Association of Vitamin D Deficiency With Diabetic Nephropathy. Endocrinol Med (Seoul) (2021) 36:106–13. doi: 10.3803/EnM.2020.826 PMC7937857 33677932

[30] de Boer IH Sachs MC Cleary PA Hoofnagle AN Lachin JM Molitch ME . Circulating Vitamin D Metabolites and Kidney Disease in Type 1 Diabetes. J Clin Endocrinol Metab (2012) 97:4780–8. doi: 10.1210/jc.2012-2852 PMC3513544 22990096

[31] Deb DK Sun T Wong KE Zhang Z Ning G Zhang Y . Combined Vitamin D Analog and AT1 Receptor Antagonists Synergistically Block the Development of Kidney Disease in a Model of Type 2 Diabetes. Kidney Int (2010) 77:1000–9. doi: 10.1038/ki.2010.22 20182412

[32] Agarwal R . Vitamin D, Proteinuria, Diabetic Nephropathy, and Progression of CKD. Clin. J Am Soc Nephrol (2009) 4:1523–8. doi: 10.2215/CJN.02010309 19478099

[33] Klaus G . Renoprotection With Vitamin D: Specific for Diabetic Nephropathy? Kidney Int (2008) 73:141–3. doi: 10.1038/sj.ki.5002693 18165810

[34] Parvanova A Trillini M Podesta MA Iliev IP Ruggiero B Abbate M . Moderate Salt Restriction With or Without Paricalcitol in Type 2 Diabetes and Losartan-Resistant Macroalbuminuria (PROCEED): A Randomized, Double-Blind, Placebo-Controlled, Crossover Trial. Lancet Diabetes Endocrinol (2018) 6:27–40. doi: 10.1016/S2213-8587(17)30359-5 29104158

[35] Krairittichai U Mahannopkul R Bunnag S . An Open-Label, Randomized Controlled Study of Oral Calcitriol for the Treatment of Proteinuria in Patients With Diabetic Kidney Disease. J Med ASSOC Thail (2012) 95:S41–47. 22619886

[36] Joergensen C Hovind P Schmedes A Parving HH Rossing P . Vitamin D Levels, Microvascular Complications, and Mortality in Type 1 Diabetes. Diabet Care (2011) 34:1081–5. doi: 10.2337/dc10-2459 PMC3114500 21525501

[37] Verroti A Basciani F Carle F Morgese G Chiarelli F . Calcium Metabolism in Adolescents and Young Adults With Type 1 Diabetes Mellitus Without and With Persistent Microalbuminuria. J Endocrinol Invest (1999) 22:198–202. doi: 10.1007/BF03343541 10219887

[38] Ahmadi N Mortazavi M Iraj B Askari G . Whether Vitamin D3 is Effective in Reducing Proteinuria in Type 2 Diabetic Patients? J Res Med Sci (2013) 18:374–7. PMC3810568 24174939

[39] Huang Y Yu H Lu J Guo K Zhang L Bao Y . Oral Supplementation With Cholecalciferol 800 IU Ameliorates Albuminuria in Chinese Type 2 Diabetic Patients With Nephropathy. PLoS One (2012) 7:e50510. doi: 10.1371/journal.pone.0050510 23209764 PMC3510062

[40] Kim MJ Frankel AH Donaldson M Darch SJ Pusey CD Hill PD . Oral Cholecalciferol Decreases Albuminuria and Urinary TGF-β1 in Patients With Type 2 Diabetic Nephropathy on Established Renin-Angiotensin-Aldosterone System Inhibition. Kidney Int (2011) 80:851–60. doi: 10.1038/ki.2011.224 21832985

[41] Bonakdaran S Hami M Hatefi A . The Effects of Calcitriol on Albuminuria in Patients With Type-2 Diabetes Mellitus. Saudi J Kidney Dis Transplant (2012) 23:1215–20. doi: 10.4103/1319-2442.103562 23168851

[42] Mehrotra R Kermah DA Salusky IB Wolf MS Thadhani RI Chu YW . Chronic Kidney Disease, Hypovitaminosis D, and Mortality in the United States. Kidney Int (2009) 76:977–83. doi: 10.1038/ki.2009.288 PMC3791220 19657329

[43] de Boer IH Ioannou GN Kestenbaum B Brunzell JD Weiss NS . 25-Hydroxyvitamin D Levels and Albuminuria in the Third National Health and Nutrition Examination Survey (NHANES III). Am J Kidney Dis (1997) 50:66–77. doi: 10.1053/j.ajkd.2007.04.015 17591526

[44] Zhang Z Sun L Wang Y Ning G Minto AW Kong J . Renoprotective Role of the Vitamin D Receptors in Diabetic Nephropathy. Kidney Int (2007) 73:163–71. doi: 10.1038/sj.ki.5002572 17928826

[45] Zhang Y Kong J Deb DK Chang A Li YC . Vitamin D Receptor Attenuates Renal Fibrosis by Suppressing the Renin-Angiotensin System. J Am Soc Nephrol (2010) 21:966–73. doi: 10.1681/ASN.2009080872 PMC2900963 20378820

[46] Diaz VA Manous AG 3rd Carek PJ Wessell AM Everett CI . The Association of Vitamin D Deficiency and Insufficiency With Diabetic Nephropathy: Implications for Health Disparities. J Am. Board Fam Med JABFM (2009) 22:521–7. doi: 10.3122/jabfm.2009.05.080231 19734398

[47] Damasiewicz MJ Magliano DJ Daly RM Gagnon C Lu ZX Ebeling PR . 25-Hydroxyvitamin D Levels and Chronic Kidney Disease in the AusDiab (Australian Diabetes, Obesity, and Lifestyle) Study. BMC Nephrol (2012) 13:55. doi: 10.1186/1471-2369-13-55 22759247 PMC3441805

[48] Taheri S Asim M al Malki H Fituri O Suthanthiran M August P . Intervention Using Vitamin D for Elevated Urinary Albumin in Type 2 Diabetes Mellitus (IDEAL-2 Study): Study Protocol for a Randomized Controlled Trial. Trials (2018) 19:230. doi: 10.1186/s13063-018-2616-5 29665833 PMC5905112

[49] Derakhshanian H Shab-Bidar S Speakman JR Nadimi H Djafarian K . Vitamin D and Diabetic Nephropathy: A Systematic Review and Meta-Analysis. Nutrition (2015) 31:1189–94. doi: 10.1016/j.nut.2015.04.009 26238534

[50] Mustafar R Mohd R Ahmad Miswan N Cader R Gafor HA Mohamad M . The Effect of Calcium With or Without Calcitriol Supplementation on Renal Function in Patients With Hypovitaminosis D and Chronic Kidney Disease. Nephrourol Mon (2014) 6:e13381. doi: 10.5812/numonthly.13381 24719814 PMC3968961

